# 2799. Evaluating Resistance Patterns of LVAD-related Pseudomonas Infections

**DOI:** 10.1093/ofid/ofad500.2410

**Published:** 2023-11-27

**Authors:** Kelly A Russo, Jeffrey Alexis, Christine M Hay, Samia H Lopa, Lu Wang, Rodolfo M Alpizar-Rivas

**Affiliations:** University of Rochester Medical Center, Rochester, New York; University of Rochester, Rochester, New York; University of Rochester School of Medicine and Dentistry, Rochester, New York; Dept. of Biostatistics & Computational Biology, Rochester, New York; University of Rochester Medical Center, Rochester, New York; University of Rochester, Rochester, New York

## Abstract

**Background:**

Left ventricular assist devices (LVADs) provide a clear benefit to patients with end-stage heart failure. Despite this, device-related infections such as driveline infections, device pocket infections, and LVAD-related bloodstream infections occur in up to 39% of implanted patients. Pseudomonas aeruginosa is a common causative organism in LVAD-associated infections and frequently develops resistance, limiting treatment options. This study aims to analyze the relationships between the frequency of acquired resistance (AR) and single-agent antibiotic courses, as well as the frequency of AR and duration of antibiotic courses; and compare the likelihood of AR between different antibiotic classes.

**Methods:**

This was a single-center retrospective cohort study involving all patients with LVAD-associated P. aeruginosa infections between 2011-2023. Patients treated with piperacillin-tazobactam, cefepime, ciprofloxacin, or meropenem, were included (n = 37). Each antibiotic course (n = 117) was associated with a pre- and post-antibiotic sensitivity. AR was defined as culture-proven, newly developed resistance to the utilized antibiotic after starting therapy.

The incidence and cumulative proportion of AR were estimated using Poisson regression and Kaplan-Meier curve analysis, respectively. A marginal proportional hazards model was used to analyze time to AR between different antibiotic groups.

**Results:**

There was a significantly lower incidence of AR with cefepime [0.8 (95% CI 0.2-3.3) per 100 person-weeks] compared to piperacillin-tazobactam [3.6 (1.7, 8)], meropenem [7.6 (4.1, 14.5)], and ciprofloxacin [6.4 (3.4, 12.5)] (p=0.04). Similarly, the cumulative proportion of AR was significantly lower with cefepime as compared to other antibiotic classes (Figure 1). The hazard rate of AR was approximately 3.5 to 6.7 times higher with each antibiotic in comparison to cefepime (p = 0.02) (Table 1).

**Figure 1.**
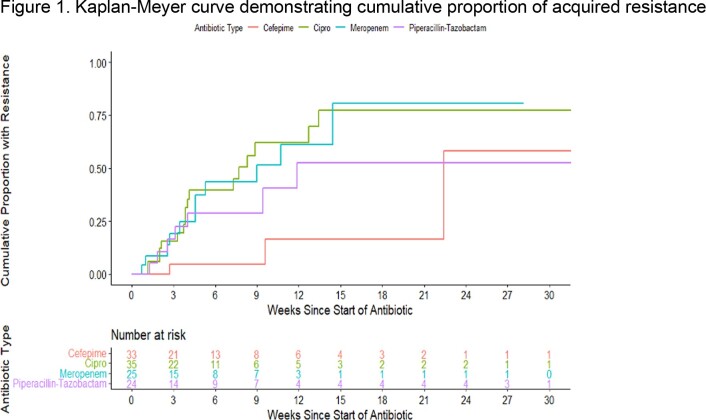
Kaplan-Meyer curve demonstrating cumulative proportion of acquired resistance

Resistance occurs early and more frequently with piperacillin-tazobactam, meropenem, and ciprofloxacin, but less frequently and late with cefepime. The cumulative proportion of acquired resistance over time differed significantly across antibiotic types (p = 0.02).

**Figure:**
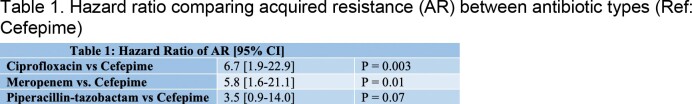

Cefepime had a statistically significantly lower hazard of AR when compared to the rest of the antibiotic groups as a whole, and when compared individually, had a significantly lower hazard than ciprofloxacin and meropenem.

**Conclusion:**

This study suggests a lower incidence of AR for LVAD-associated P. aeruginosa infection treated with cefepime. AR also seems to develop later in patients treated with cefepime. There is a 3.5-6.7-fold increase in the risk of acquiring resistance with other antibiotics compared to cefepime.

**Disclosures:**

**Jeffrey Alexis, MD**, Abbott: Grant/Research Support

